# *Bacillus anthracis* Phylogeography: Origin of the East Asian Polytomy and Impact of International Trade for Its near Global Dispersal

**DOI:** 10.3390/pathogens14101041

**Published:** 2025-10-14

**Authors:** Gilles Vergnaud, Markus H. Antwerpen, Gregor Grass

**Affiliations:** 1 Institute for Integrative Biology of the Cell (I2BC), University Paris-Saclay, 91198 Gif-sur-Yvette, France; 2 Bundeswehr Institute of Microbiology (IMB), 80937 Munich, Germany; markusantwerpen@bundeswehr.org (M.H.A.); gregorgrass@bundeswehr.org (G.G.)

**Keywords:** anthrax, Asia, China, Germany, Bengal, evolution, genomics, phylogeography, wgSNP, infectious disease

## Abstract

*Bacillus anthracis* is the etiological agent of the zoonotic disease anthrax. The pathogen has colonized many regions of all inhabited continents. Increasing evidence points to a strong contribution of anthropogenic activities (trade) in this almost global spread. This article contributes further genomic data from 21 *B. anthracis* strains, including 19 isolated in Germany, aiming to support and detail the human role in anthrax dispersal. The newly sequenced genomes belong to the *B. anthracis* lineage predominant in China. This lineage is remarkable because of its phylogenetic structure. A polytomy with nine branches radiating from a central node was identified by whole-genome single-nucleotide polymorphism (wgSNP) analysis. Strains from Germany populate two among the nine branches. Detailed analysis of the polytomy indicates that it most likely emerged in China. We propose that the polytomy is the result of the import of contaminated animal products in a limited spatiotemporal frame, followed by the distribution of these products to different locations within China, where new *B. anthracis* lineages then became independently established. Currently available data point to Bengal as a likely geographic source of the original contamination, and the history of trade exchanges between Bengal and China agrees with the early fifteenth century as a likely time period. The subsequent exports to Germany would have occurred during the 19th century according to German trade history. Notably, Germany has been experiencing localized anthrax outbreaks from this trade heritage up into the 21st century.

## 1. Introduction

Anthrax, the zoonotic disease caused by the bacterial species *Bacillus anthracis*, is present in various regions of each permanently inhabited continent. Active endemic areas are located in most of Asia and Africa, parts of the Americas, Australia, and Europe [1]. *B. anthracis* is highly monomorphic, and DNA-based typing methods, including Multiple-Locus Variable-Number of Tandem Repeats (VNTR) Analysis, also known as MLVA, were essential to start resolving its population structure [2,3,4,5,6]. The emergence of whole-genome sequencing (WGS) allowed for the demonstration that the species was derived from the clonal expansion of a single ancestral *Bacillus cereus* strain carrying two virulence-associated plasmids, pXO1 and pXO2 [5,7,8,9]. WGS data analysis also showed that the average nucleotide identity (ANI) values between any two strains of *B. anthracis* are higher than 99% [10].

Clonality implies that the species emerged at a precise time point and geographic location. An African origin for *B. anthracis* was proposed based on the genetic diversity of lineages observed in Africa [11]. At the time of that proposal, two main lineages had been described using MLVA, the so-called A and B lineages, and both were present in southern Africa. However, in subsequent years the very rare lineage C defining the most ancestral node within the *B. anthracis* phylogeny was identified in North America, where lineages A and B are also present today, most likely recently introduced from other regions worldwide [9]. In addition, the phylogeny of the B lineage has since been described in more detail, demonstrating that the A and B lineages coexist in many areas across Eurasia. More importantly, currently available genetic data regarding the B lineage indicate that it was introduced from Eurasia to South Africa only after the development of long-distance maritime trade routes along the African coast [12,13,14,15,16]. Consequently, the previously established belief that high genetic diversity can indicate the geographic origin of *B. anthracis* appears to be of limited value.

A more recently proposed hypothesis in favor of an African origin of *B. anthracis* uses non-*B. anthracis* outgroup organisms for the anthrax virulence plasmids and for the chromosome, respectively. This hypothesis results from the finding in the African rainforest (central Africa) of the closest relatives of *B. anthracis* virulence plasmids in *B. cereus* strains [17,18,19,20,21,22,23,24,25] and also of the closest known *B. cereus* chromosome [26]. Strain 2000031002, isolated in the Democratic Republic of Congo, is unique in the *B. cereus* multi-locus sequence typing (MLST) database by its sequence type (ST130) differing from the nearest *B. anthracis* strains by a single base-pair among the seven loci used in the MLST assay covering 2600 bp or 0.5% of the *B. anthracis* genome [27].

Important for the understanding of the *B. anthracis* species phylogenetic structure, some lineages present almost exclusively in Africa have been described. These African lineages were initially identified by MLVA and called “D” and “E” [28]. The “E” branch corresponding to West African strains was also called Aβ [29,30,31]. Whole-genome sequencing allowed researchers to precisely position the monophyletic African clade within *B. anthracis* phylogeny [23,32,33,34,35,36,37]. This African clade is now designated as “Ancient A” in the current canonical SNP (canSNP) nomenclature [34].

Based on the “Central Africa” geographic rooting hypothesis, and on the description of the African clade, the topology of the phylogenetic tree of *B. anthracis* would indicate that lineages C, B, and A represent three successful “Out of Central Africa” exports, in this chronological order [23,38]. In this model, the Nile itself (or traffic on or along the Nile) might have been the carrier of the contamination from the Central Africa cradle. Accessorily, this model provides a parsimonious explanation for the relative frequency of the three lineages and predicts that Middle East and more precisely Egypt was the initial geographic root in the Out-of-Africa process.

The dating of these exports is not established. Most ancient literature available regarding the disease caused by *B. anthracis* is ambiguous, as anthrax has been confused with other diseases until the end of the nineteenth century [39,40,41,42,43]. For instance, although Philibert Chabert is often credited for providing the first description of anthrax as a specific disease in the year 1780, he still considered “fièvre charbonneuse”, “charbon essentiel”, and “charbon symptomatique” to be different expressions of the same disease [44,45]. Another century was needed to show that the last two diseases were caused by *Clostridium chauvoei* [40]. The description of the fifth plague of Egypt dated approximately 3500 years before present (ybp) has prompted some authors to suggest that it was anthrax, essentially on the basis of the spectrum of animal species that were affected [41]. A different example given by Vergil [46] is much more conclusive [47]. Vergil, writing approximately 2000 ybp, was referring to an “ancient event”, which occurred in “Noricum” and along the Timavo river, i.e., within modern Austria and Slovenia [48,49]. This very well described “anthrax” is a disease present in the Alps, able to cause sudden death in different species of domestic animals. It is accompanied by hemorrhage from the orifices, and is able to infect humans by contact with animal products. Interestingly, the text indirectly implies that this “anthrax” was not known in nearby Italy 2000 ybp.

The availability of WGS data from a few representative strains allowed researchers to propose a first tentative dating for the Most Recent Common Ancestor (MRCA) of *B. anthracis* [5,50,51,52]. The MRCA dating estimate of 13,000–50,000 ybp categorized *B. anthracis* among the young pathogens group [51]. The authors proposed that within *B. anthracis*, lineage A would have radiated during the Neolithic largely because of anthropic factors including trade of contaminated animal products. Their model was not compatible with the whole of *B. anthracis* having radiated during the Neolithic since this would stand in conflict with their proposed MRCA dating. More so, the *B. anthracis* species could be much older than its MRCA, with early lineages gone extinct without any trace. Of note, the most basal extant *bona fide B. anthracis* lineage, the C branch (C.Br.), comprises only very few members recovered in North America from environmental samples [6].

The dating of the emergence of *B. anthracis* is notoriously difficult due to its ecology. Between two cycles of infection, the bacteria may stay in the environment as inert spores. In this very resilient resting stage, the organism is able to survive for a number of years or even decades depending upon the environmental conditions [1,36,53,54,55]. As a result, the molecular clock is not ticking at the same pace across the entire *B. anthracis* phylogeny. The previously proposed MRCA estimate was based upon an empirical evaluation of 0.28 cycles of infection per year, a value suggested by observations in Canada during the recent past decades [50,56]. This estimate is likely to be highly variable and dependent on the ecological context. The most ancient robust dating point currently available along the phylogeny of *B. anthracis* is positioned within the A branch. It was inferred from combining phylogenetic analysis and historical events. The predominant North American lineage, called Western North America (WNA), was most likely imported from Western Europe, during the 16th or 17th century instead of 10,000 years ago from Asia as proposed earlier [23,32,33,50,57,58]. The speed of evolution of *B. anthracis* after this introduction into North America has been at least ten-fold higher than the speed of evolution of its progenitor lineage in Europe. This has been interpreted to reflect the pathogen’s arrival in an anthrax-naïve ecosystem. There, the rampant spread of the disease was able to sustain high numbers of infection cycles, likely more than one per year [5,23,50,57]. More recently dated introductions of anthrax into naïve ecosystems are consistent with this view. For instance, Australia is considered to have been contaminated with *B. anthracis* via bones imported from India in 1847 [59]. Likewise, multiple contamination events due to the import of Kashmir wool were reported starting in the 19th century in the United Kingdom, a country which seems to have been anthrax-free before that time [43,60,61,62].

In complement to these intra-species dating points, the availability of close genetic neighbors for both the virulence plasmids and chromosome allowed researchers to establish that the species ancestor is approximately twice as ancient as the MRCA of extant *B. anthracis* clades in terms of genetic distance. The first estimate was deduced from the phylogenetic analysis of the pXO1 plasmid and of its homologs in *B. cereus* strains causing anthrax-like disease in the USA and in *B. cereus* biovar *anthracis* strains from Central Africa [23]. A second, independent estimate was based on the study of ratios of non-synonymous to synonymous substitutions (dN/dS ratios) within *B. anthracis* compared to its nearest neighbors. Whereas high dN/dS values were measured within *B. anthracis* as expected for a recent and clonal species, the phylogenetic branch located immediately upstream of the current *B. anthracis* MRCA (predicted to partly belong to *B. cereus*) showed an intermediate value interpreted as an indication that clonality predates the MRCA of *B. anthracis.* This allowed researchers to estimate the genetic distance between the MRCA and the ancestor [22].

These new dating estimates opened the possibility that the emergence of the whole *B. anthracis* species, and not just the A clade, might have occurred during the Neolithic period via anthropic factors. The arrival of pastoralism in the vicinity of the African rainforest approximately 5000–7000 ybp would have triggered this emergence [23,63]. Under the Neolithic model, the B clade would have emerged out of Africa 1500–3000 ybp. Intriguingly, this tentative dating and the current geographic distribution of the B clade, present in the Alps but not in the rest of Italy, make it a candidate for being responsible for Vergil’s Noricum outbreak [13,15,32]. The C clade might be the remnant of an even earlier wave of *B. anthracis* dissemination.

Emergence of the most widespread and frequent A clade 500–1500 ybp predicts that the phylogeny of its sub-lineages might be understood in view of relatively recent historical events [23]. The existence of a limited number of major sub-lineages suggested by the early genotyping methods was confirmed by WGS data [2,5,7,34,64,65,66]. The A clade is divided into TEA (TransEurAsia), Australia94, Vollum, V770, WNA (Western North America), and Sterne/Ames [34]. The Ancient A lineage today included in the A clade, although initially called D and E, has a particular status in the Neolithic model because of its strong African association [23,36]. This association makes it the only likely candidate for being the modern representative of the initial ecotype [38].

The geographic rooting of sub-lineages is uncertain. Strains isolated in Türkiye contribute the shortest and rarest branches within the TEA polytomy, which may constitute indirect evidence for a geographic rooting of TEA in the Middle East [15,34]. Australia94 is predominant in Caucasia and strains from this region and from Türkiye currently contribute the shortest branches in this lineage’s phylogeny [34,67]. The Vollum lineage is most likely associated with Central Asia and is the main contaminant of Kashmir wool [15,62,68]. The V770 lineage is predominant in South America but given our current understanding of the phylogeography of *B. anthracis*, this location is not likely to be the source location, which is consequently currently unknown [55].

The Sterne/Ames lineage is split into canSNP sub-clades A.Br.075(Sterne) and A.Br.081(Ames) following the nomenclature defined by [34]. A.Br.075(Sterne) contains the Sterne 34F2 vaccine strain developed almost one century ago in South Africa from a strain recovered in the course of major outbreaks [69]. The lineage shows a strong association with Bangladesh. The most parsimonious explanation for the wide geographic distribution of this particular lineage is long-distance trade from the gulf of Bengal, where for instance, both Denmark and the Netherlands were established in the 17th and 18th centuries [54,70,71,72]. A.Br.081(Ames) contains the so-called Ames strain associated with the 2001 anthrax-laced bioterrorism letters [73]. The A.Br.081(Ames) sub-lineage is structured as a polytomy with nine branches, reminiscent of the TEA lineage with its seven branches [12,13,14,15]. Strains from the A.Br.081(Ames) polytomy have also been recovered from multiple countries including Europe [5,33,54,72,74,75,76,77,78]. The Ames branch itself is strongly associated with China and Central Asia [15,74,79,80]. The eight other branches are not as well described. The most recent global canSNP nomenclature published in 2016 covered four among the nine branches known today [34].

The present report is focused on the A.Br.Sterne/Ames lineages. We sequenced 19 archival strains isolated in Germany, one isolated in Switzerland and one isolated in China, all 21 strains assigned to the A.Br.081(Ames) polytomy. We analyzed and discussed their phylogenetic position compared to publicly available data by whole genome SNP (wgSNP). We interpret available data as showing that China was contaminated within a very limited timeframe, possibly from Bengal in the early fifteenth century in the common era (CE), and subsequently contaminated a number of European countries and other regions.

## 2. Materials and Methods

### 2.1. Strain Collection, DNA Extraction, and Whole-Genome Sequencing

*B. anthracis* isolates from our archival strain collection were grown on Columbia blood agar (Becton Dickinson, Heidelberg, Germany) or trimethoprim–sulfamethoxazole–polymyxin blood agar (TSPBA) [1,81] and chemically inactivated with 4% (*v*/*v*) Terralin PAA (Schülke and Mayr GmbH, Norderstedt, Germany) [82] at the Bundeswehr Institute of Microbiology biosafety level 3 (BSL-3) facility. Genomic DNA was isolated using a MasterPure™ Gram-Positive DNA Purification Kit (Lucigen, Middleton, WI, USA). DNA concentrations were quantified using a Qubit dsDNA HS Assay Kit (Thermo Fisher Scientific, Dreieich, Germany) according to the supplier’s protocol, and DNA was stored at −20 °C until further use.

Genomic libraries of *B. anthracis* DNA were constructed using a NEBNext^®^ Ultra™ II DNA Library Prep Kit (New England Biolabs, Frankfurt am Main, Germany) or Illumina DNA Prep (Illumina, Berlin, Germany) with 100 ng of input DNA. Subsequent use of the Illumina MiSeq platform with 2 × 300 bp v3-chemistry produced at least 300,000 reads for each isolate.

The sequence data generated are publicly available in the NCBI-EBI Sequence Read Archive (SRA) repository, Bioproject PRJNA309927, SRA accession numbers SRR34470768 to SRR34470788 (Supplementary Table S1).

### 2.2. Whole-Genome SNP Analysis

Publicly available assemblies and short-read sequence read archives (SRAs) were downloaded via EBI-ENA (last updated 14 April 2025). Raw reads were assembled using SKESA [83]. Assemblies were split into 50 bp long artificial reads that were then used for SNP calling by mapping on the Ames ancestor reference genome (assembly accession GCF_000008445.1) [33,84]. BioNumerics version 8.1 (Applied-Maths, Sint-Martens-Latem, Belgium) was used for SNP calling as previously described [85]. Lineage assignments followed the global nomenclature based on selected SNPs with the exception of the A.Br.048 lineage (alias A.Br.005/007 [5]), which was kept separate from A.Br.007(Vollum) due to its deep branching and very different geographic distribution [34]. BioNumerics was used for maximum parsimony analysis and dendrogram drawing. Trees were rooted using a representative from the nearest neighbor lineage as an outgroup. The WGS dataset with the lowest “nb_unknown_bases” value indicated in Supplementary Table S1 was selected as an outgroup among available strains.

## 3. Results

**Figure 1:**
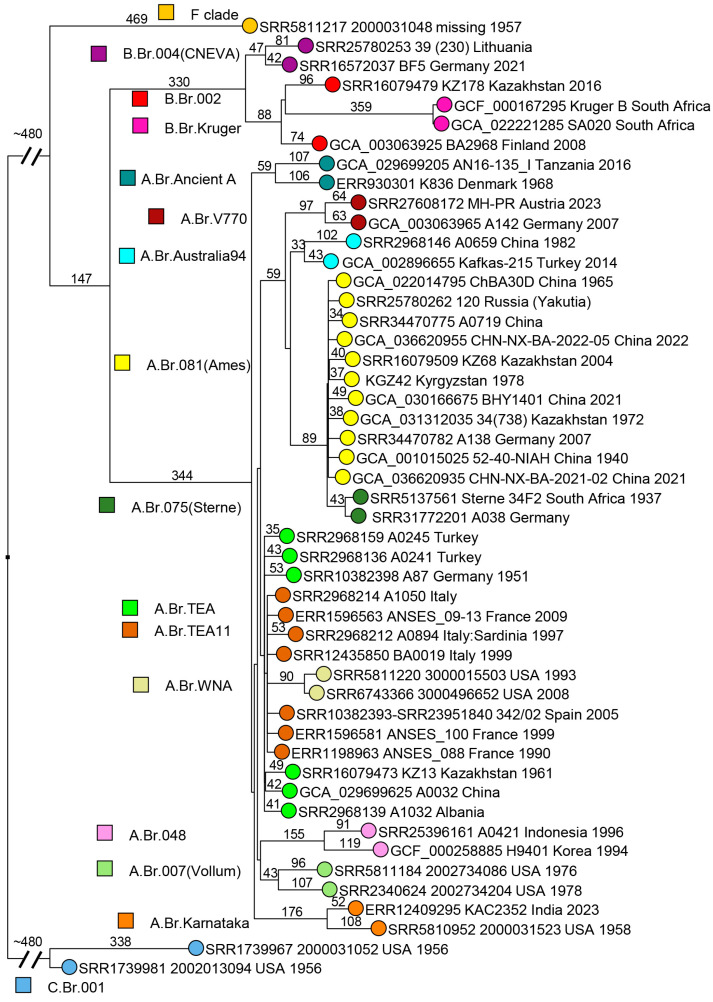
Update of *Bacillus anthracis* global phylogeny. Forty-nine entries allowed defining the ancestral node within each *B. anthracis* lineage [34]. When more than one strain was available, the strain defining the shortest branch was retained. Polytomies are represented by one strain per radiating branch. Mapping against the Ames ancestor reference genome (assembly accession GCF_000008445.1) allowed for the calling of 6262 SNPs. The maximum parsimony tree has a size of 6289 SNPs, corresponding to a homoplasy level of 0.4%. The position of the root was estimated using *B. cereus* strain BC38B (genome accession GCA_025946485) as an outgroup [86]. Branch lengths >30 SNPs are indicated. Strains are colored according to lineage as indicated, and labels include accession number, strain ID, country of isolation, and year of isolation, when known.

Two thousand and four hundred WGS datasets were downloaded from public repositories, including assemblies and short-read archives (SRAs) and confirmed as bona fide *B. anthracis* (Supplementary Table S1). All *B. anthracis* chromosomes could be assigned to previously defined clades [5,23,34]. More than nine hundred were duplicates, most frequently corresponding to WGS datasets deposited as assemblies and SRAs, to reference or vaccine strains sequenced independently by different laboratories or to genetically modified strains. In order to produce an overview of the global phylogeny, we selected strains defining the most ancestral node within each clade. In cases where more than two branches radiated from the most ancestral node (polytomies), we included one strain per branch. When more than one strain was available, we retained the strain contributing the shortest branch. Forty-nine strains were selected through this process. Figure 1 shows the resulting wgSNP phylogenetic tree. The position of the root was established using the WGS data from strain BC38B, genome accession GCA_025946485, which is the currently available *B. cereus* chromosome genetically closest to *B. anthracis* [86].

The C clade defines the shortest branch in terms of number of SNPs, followed by the F branch represented by a unique strain [23]. The B clade is split into two, B.Br.004(CNEVA) and B.Br.002, including B.Br.Kruger in the current canSNP nomenclature [34]. The shortest branch within the Western European B.Br.004 has a length of 89 SNPs, whereas the shortest branch within Eurasian B.Br.002 has a length of 162 SNPs. The branch represented by the strain from Finland belonging to B.Br.002 is also populated by strains from Western Siberia [14]. Finland could have possibly been contaminated from Russia in 1719 [87]. Only two strains from Finland have been described so far, one from the A clade, A.Br.064(V770), found in numerous countries and the one from the B clade [88]. The Finnish terminal B clade branch is twice as long as that of the closest Western Siberian strains [14].

Within the A clade (excluding Ancient A), a new node is defined in basal branch A.Br.005 by strains from Karnataka, India, in addition to one strain from the USA. The USA strain (biosample SAMN07332902, Supplementary Table S1) was isolated in 1958, New Hampshire, from imported goat hair, and the WGS data was made public in 2017 [23,35]. The Karnataka strains were isolated in 2018–2023, and sequence data were deposited in 2024 by the Indian Biological Data Center. Consequently, this lineage was not defined in the canSNP scheme published in 2016 [34], and we propose to call it “A.Br.Karnataka”. The shortest branches within the A clade are contributed by the TEA and the TEA11 polytomies. Two TEA branches are defined by only one and two strains, respectively, all from current Türkiye [34]. A third branch gave birth to the TEA11 polytomy, also with seven branches [23,57]. The North American WNA clade emerged from one of the TEA11 branches and is recognized by its characteristic long branch compared to the rest of TEA and TEA11 [23,57]. Under the current interpretation of the phylogeography of *B. anthracis*, the TEA polytomy might have emerged from the Middle East during the 14th–15th century CE with the then Ottoman Empire as the current best candidate for the original location, mindful of the caveat that only few data are available from a number of neighboring countries as alternative origins [23].

**Figure 2:**
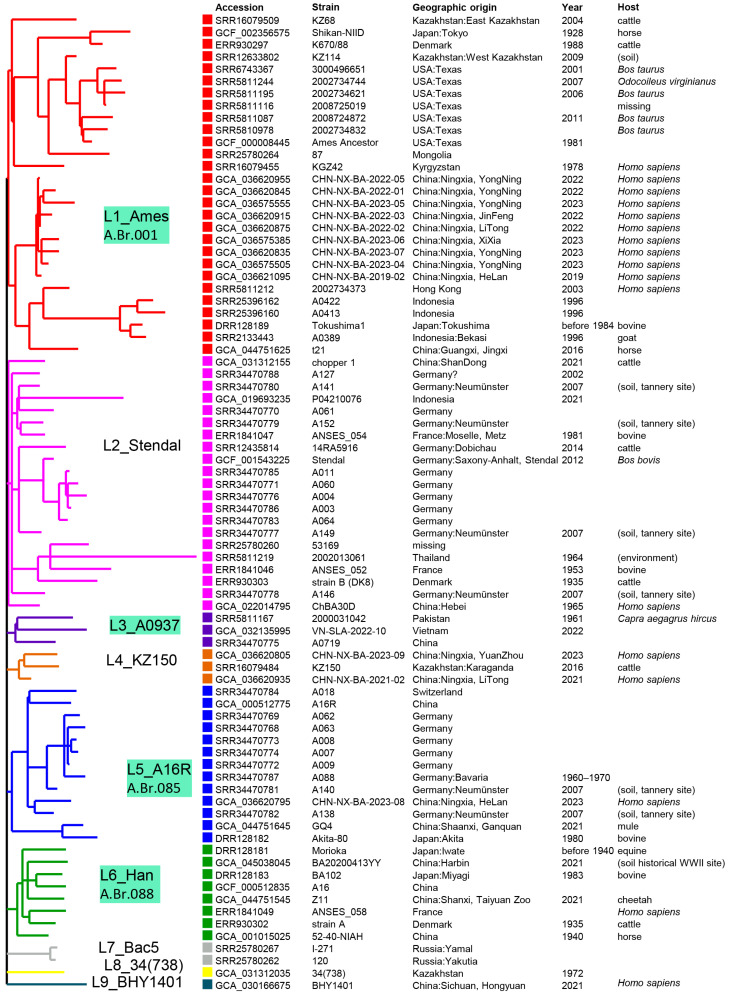
The nine-branches polytomy within A.Br.081(Ames). Rooted maximum parsimony tree deduced from wgSNP analysis of 80 *B. anthracis* entries. A total of 1707 SNPs were called (Table S2), and the tree size is 1716 (homoplasia 0.5%). Branches are colored according to their branch assignment as indicated. WGS accession number, strain ID, geographic origin, year of isolation, and host are indicated if known. The four branches previously identified in the 2016 canSNP nomenclature [34] are highlighted in green and the canSNP assignments are indicated.

The Sterne/Ames polytomy is represented by 447 public WGS datasets, not including the 21 datasets contributed here. Two hundred and ten genomes group within branch A.Br.075(Sterne) [54] whereas 237 belong to branch A.Br.081(Ames) and contribute to the nine other branches including the Ames lineage. Eighty-eight among these 237 genomes are duplicates, 42 are derived from the Ames ancestor reference strain, 28 are redundant (identical wgSNP genotype and country of origin), and two datasets have insufficient quality (low coverage). Two datasets available as assemblies were discarded because they constituted suspiciously long terminal branches. Sixteen WGS datasets were not included because their wgSNP genotype had a distance of less than five SNPs from another one from the same country. Eventually, 59 public WGS datasets corresponding to distinct wgSNP genotypes were used in subsequent analyses, together with the 21 newly sequenced strains (Supplementary Table S1). Figure 2 shows the result of a rooted maximum parsimony tree analysis of the 80 strains, colored according to branch assignment within the polytomy. The nine branches L1-L9 are named according to one of the assigned strains. Four branches were defined in the canSNP nomenclature [34], L1_Ames (containing A.Br.001 as most basal branch), L3_A0937 (defined by a single strain in [34]), L5_A16R (containing A.Br.085 as the most basal branch), and L6_Han (containing A.Br.088 as the most basal branch). Strain A0389 was identified as potentially defining a branch within the polytomy [34]; however, a chromosomal SNP at position 1,797,708 defines an established basal branch allowing to formally assign this branch and strain A0389 to L1_Ames (thus, SNP 1,797,708 in Table S2 belongs to A.Br.001 instead of A.Br.002 as indicated in [34]).

**Figure 3:**
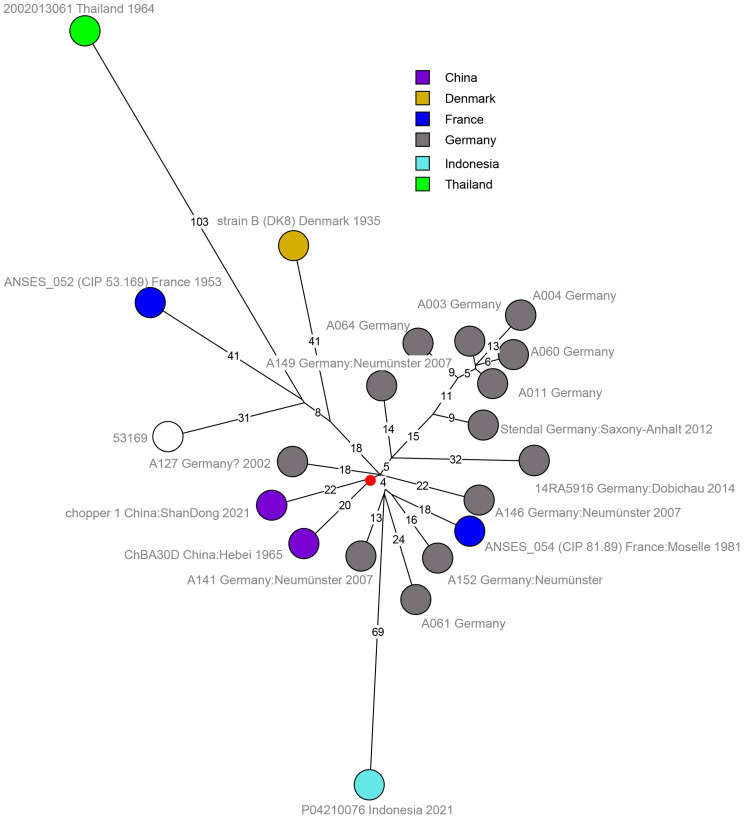
Detailed SNP structure of the L2_Stendal branch. A total of 21 *B. anthracis* strains were retained after removal of duplicates and strains with identical wgSNP genotypes and geographic origins. The maximum parsimony tree deduced from 597 SNPS had a size of 598 (homoplasia 0.2%). Strains are labelled with strain ID, geographic origin, and year of isolation if known. Branch lengths of more than two SNPs are indicated. The red dot shows the position of the root of the L2_Stendal branch. This root is located at a distance of one SNP from the root of the nine branches of the A.Br.081(Ames) polytomy.

The shortest length from the root (i.e., the center of the polytomy) to the tips is observed within the L4_KZ150 branch. Strain CHN-NX-BA-2021-02 from Ningxia Hui autonomous region (China) was at a distance of 15 SNPs from the root. The shortest branches in lineages L1_Ames were also represented by Chinese strains from Ningxia [89]. The 19 newly sequenced German strains could be assigned to L2_Stendal (11 strains) or L5_A16R (8 strains). German and Chinese strains exhibited the shortest branches within L2_Stendal (Figure 3). The German strains designated “Neumünster” were isolated from an abandoned historic tannery site.

**Figure 4:**
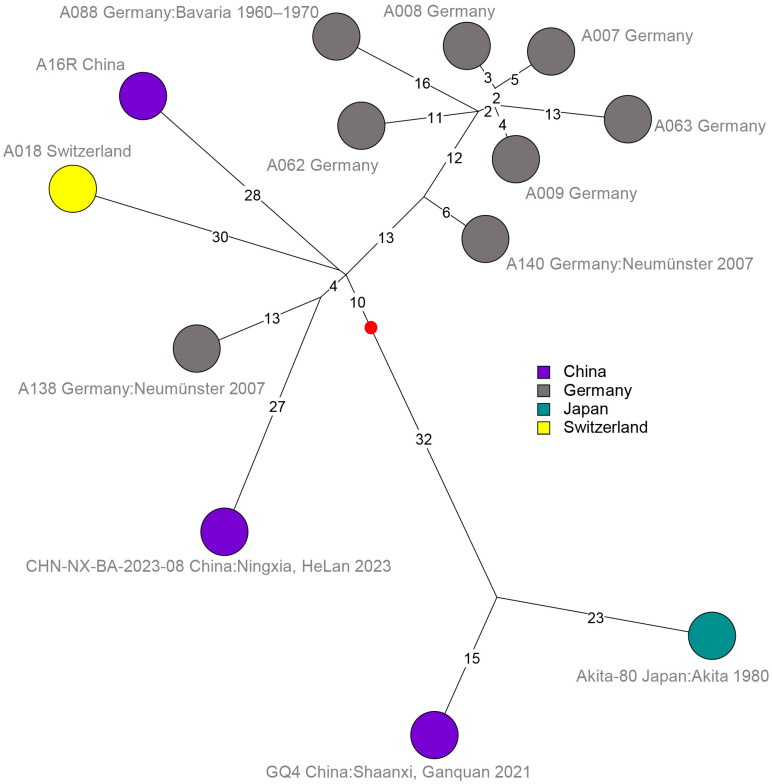
Detailed SNP structure of the L5_A16R branch. Thirteen *B. anthracis* strains were retained after removal of duplicates and strains with an identical wgSNP genotype and geographic origin. A total of 270 SNPs were called, and the maximum parsimony tree size was 270 (no homoplasia). Strains are labelled with strain ID, geographic origin, and year of isolation if known. Branch lengths above one SNP are indicated. The red dot shows the position of the root of the L5_A16R branch as determined using the Ames ancestor strain as outgroup. This root is located at a distance of three SNPs from the root of the nine branches of the A.Br.081(Ames) polytomy.

Isolates from the historical German tannery site in Neumünster also yielded the shortest branches in L5_A16R (Figure 4). Importantly, strains from China appeared to populate lineages L1 to L6 and L9. The very rare lineages L7_Bac5 and L8_34(738), populated by two strains from Russia and one strain from Kazakhstan, respectively, were the only ones in which Chinese strains were currently absent.

The longest branches in the A.Br.081(Ames) polytomy were associated with strains from Thailand (L2_Stendal), Indonesia (L2_Stendal and L1_Ames), and Japan (L1_Ames) (Figure 2 and Figure 3). These observations further illustrate the distortion in the molecular clock and temporal signal existing within *B. anthracis* evolution. The German strains investigated herein do not appear to be monophyletic, i.e., they are interspersed with strains from China or other Asian countries. This is illustrated in the L5_A16R branch (Figure 4) with strain A138 clearly separated from the other German isolates, and this makes sense insofar as these strains originate either from abandoned German tanneries or secondary outbreaks likely associated with imported goods.

**Figure 5:**
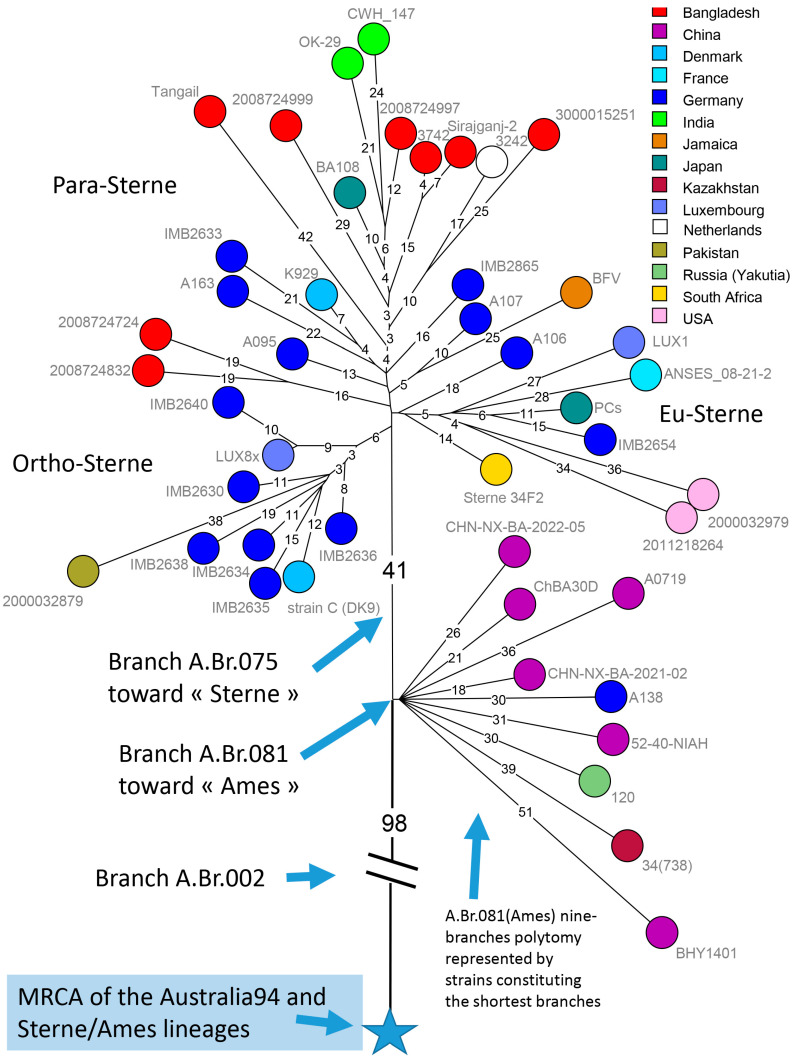
Structuring the A.Br.Sterne/Ames lineage based on a proposed geographic rooting in Bengal. Forty-five representative strains with a known geographic origin were selected for wgSNP analysis. Thirty-six belonged to the Sterne lineage. The last nine represent the L1 to L9 branches of the A.Br.081(Ames) polytomy displayed clockwise. Within each branch, the strain closest to the root of the polytomy was selected. One strain from A.Br.Australia94 and one strain from A.Br.V770, the closest outgroups, were used to root the Sterne/Ames lineage. The blue star shows the position of the MRCA of the Australia94 and Sterne/Ames lineages, and defines the start of the Sterne/Ames lineage. A total of 1198 SNPs were called within the Sterne/Ames lineage, the maximum parsimony tree has a size of 1199 (homoplasia 0.09%). Branch lengths above two SNPs are indicated. The defining A.Br.081 “branchlet” separating the A.Br.081(Ames) polytomy from A.Br.075(Sterne) has a length of just one SNP. It corresponds to the canSNP at chromosome position 515,111 of the Ames ancestor reference genome [34]. The positions of the A.Br.002 and A.Br.075 branches are shown [34]. Strains are colored according to geographic origin and labelled with strain names. The nick-naming of A.Br.075(Sterne) sub-lineages proposed by [54] (Ortho-Sterne, Eu-Sterne, Para-Sterne) is indicated.

The A.Br.075(Sterne) lineage is the immediate genetic neighbor of the A.Br.081(Ames) polytomy. A.Br.075(Sterne) is strongly associated with Bangladesh [54,72]. Figure 5 shows a representation of the Sterne/Ames topology, rooted using the A.Br.Australia94 lineage. Starting from the blue star which represents the MRCA of Sterne/Ames and of its nearest neighbor Australia94, the lineage expands for 98 SNPs, corresponding to the A.Br.002 branch, until a first node is encountered. This node is located one SNP away from the root of the nine-branches polytomy including L1_Ames. This canonical SNP defines the A.Br.081 branch [34]. The A.Br.075(Sterne) lineage expands for 41 SNPs, constituting the A.Br.075 branch, until new nodes are reached. The first two associated sub-lineages, now nicknamed Ortho-Sterne and Eu-Sterne, are populated by strains isolated in Europe, but also Japan, South Africa, Pakistan, or the USA [54]. No strains from Bangladesh are present, but the geographic spread suggests that these lineages are not monophyletic, i.e., are not the result of a single export. For instance, the Eu-Sterne strain from Japan has a European strain as a closest relative, but it is unlikely that the European strain was imported from Japan or that the Japanese strain was imported from Europe [16,54,90]. The most parsimonious interpretation of this observation is that Ortho-Sterne and Eu-Sterne are now extinct in Bangladesh but have been preserved by their exports, or that the origin regions in Bangladesh have not yet been sampled.

**Figure 6:**
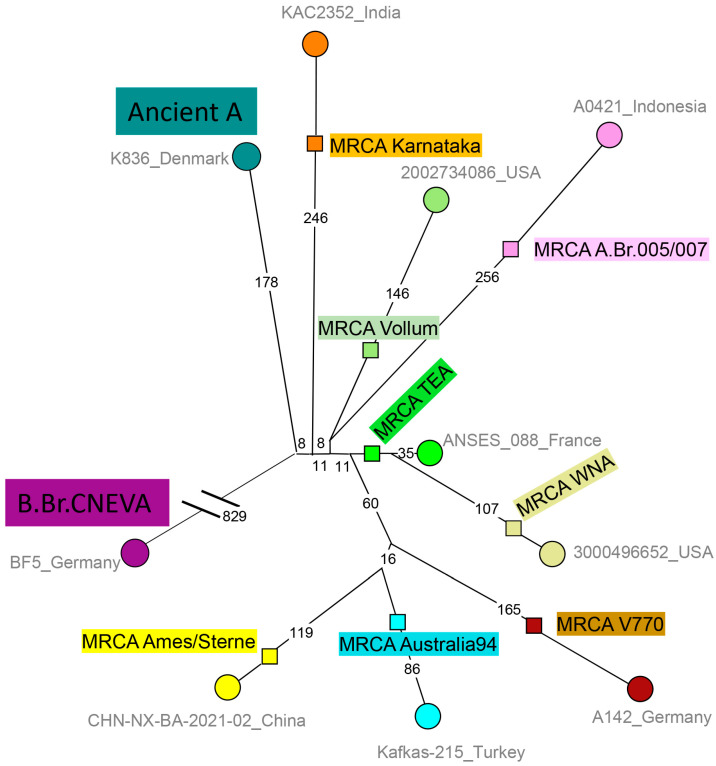
Schematic representation of the *B. anthracis* A clade. Clade A chromosomal data from Figure 1 were extracted keeping only the shortest branches within each of the main sub-lineages, and showing the position of each hypothetical MRCA (squares). A total of 2279 SNPs were called, and the tree size is 2285 SNPs (homoplasia 0.27%). The color codes reflect lineage assignments. A representative from both Ancient A and the B clade, B.Br.CNEVA (broken line) are included as outgroups. The size (number of SNPs) of the branches is indicated. Strain IDs and country of isolation are shown.

Figure 6 provides a simplified view of the A clade. This view illustrates that the TEA lineage is by far the shortest lineage within the A clade. As previously noted, Australia94 is not yet strongly geographically rooted. It is the nearly exclusive lineage present in Georgia [67], and strains from Georgia and Türkiye currently contribute the shortest branches within Australia94. Notably, only limited data are available regarding *B. anthracis* from most countries in the Middle East, so that no firm conclusion can be drawn regarding the more precise geographic routing of Australia94 to the Middle East. More generally, the Middle East appears to be a likely geographic root for the A clade, featuring A.Br.Karnataka (Southern India), A.Br.Vollum (Northern India–Pakistan), A.Br.Sterne (Bangladesh), A.Br.Australia94 (Georgia, Türkiye, and India), and A.Br.TEA (Türkiye).

## 4. Discussion

In the present report, we contribute sequence data from 21 archival *B. anthracis* strains assigned to the widespread A.Br.081(Ames) lineage. Nineteen strains originated from Germany, particularly from an abandoned tannery site processing imported or local hides [91]. We could show that these strains clustered within two of the nine branches constituting the A.Br.081(Ames) polytomy. Importantly, seven of the nine branches are populated by strains isolated in China, and the two exceptions are branches comprising at present only one or two strains.

These observations strongly suggest that the A.Br.081(Ames) polytomy emerged in China, with Northeastern China and the provinces surrounding Beijing as the current best candidate location. Investigations of the genetic diversity of *B. anthracis* in China have previously shown that A.Br.Vollum, A.Br.Australia94, A.Br.TEA, and A.Br.081(Ames) are present in this country [74,92]. More precisely, Vollum, Australia94, and TEA were only found in the Xinjiang autonomous region in Western China, whereas A.Br.081(Ames) strains were present in the rest of China.

A polytomic structure such as the one observed within A.Br.081(Ames) is a topology expected if animal products causing an outbreak have been exported and distributed. Subsequently, independent lineages derived from the common progenitor will have the opportunity to expand in each location contaminated with these exported animal products. The fact that the contaminating incident created a polytomy suggests that this event was the result of imports within a short timeframe and restricted geographic origin. Previous reports investigating anthrax outbreaks showed that initial polytomies are quickly lost by genetic drift. Three recent outbreaks occurred in France in a timeframe of 12 years, in the same geographic area. Sequence analysis of the 32 *B. anthracis* strains recovered from distinct animals showed that the polytomy emerging in the first two outbreaks was already lost in the third [23]. An export of strains from the third outbreak would have contributed to only one branch of the polytomy emerging in outbreaks 1 and 2. Consequently, the most parsimonious interpretation of available data regarding the phylogeography of *B. anthracis* in China (not including Xinjiang) is that China was contaminated in a very short timeframe by imported animal products, such as hides, coming from Bengal. There is currently no evidence for the presence of more ancient *B. anthracis* lineages in China, so that China might have been anthrax-free before that time. The contamination event would have occurred before the 17th century, when Europe became increasingly contaminated in turn via the emerging long-distance maritime routes including trade with both Bengal and China. Notably, there has been one instance of intense diplomatic exchanges between Bengal and China, during the period of the Chinese maritime expeditions from 1405 to 1433 with no comparable ventures before or after that period [93]. These diplomatic exchanges included tributes sent from Bengal to the then capital of China, Nanjing (or Beijing after 1421). This limited timeframe would be compatible with the export of a polytomy.

In contrast, the A.Br.Sterne/Ames distribution pattern observed in Germany and other European countries is best explained by regular trade continuing for decades, introducing a diversity of *B. anthracis* from already contaminated countries, and thus a variety of sub-lineages may be recovered later as illustrated here and in previous reports [54]. These continued contaminations in Europe have been made possible by long-distance maritime trade of animal products. Consequently, such trade actions must have started during or after the 17th century, and lasted until the early 20th century when control measures against *B. anthracis* contamination were implemented in many European countries [55].

Only limited *B. anthracis* WGS data are available from other Asian countries. The western neighbor of China, Kazakhstan, has been shown to be predominantly contaminated by strains from the TEA lineage [15]. Data from Japan indicate that the Japanese archipelago has been contaminated by *B. anthracis* strains from three branches of the A.Br.081(Ames) polytomy (L1_Ames, L5_A16R, and L6_Han), in addition to Para-Sterne and Australia94, typical of recent imports [94]. Similarly, Vietnam has been shown to be contaminated by L3_A0937 and by derivatives of the Pasteur II vaccine strain [95,96]. The Pasteur vaccine comprised two *B. anthracis* strains, one of which, Pasteur II, still virulent. The Pasteur vaccine was introduced in Vietnam by Alexandre Yersin in 1911 [97]. This partial coverage of the *B. anthracis* diversity in (East) Asia supports our prediction that most of these regions might have been anthrax-free until the last two or three hundred years, at least as far as current data suggest. Precise investigations within Chinese provinces, as recently conducted in the Ningxia Hui autonomous region, might eventually allow reconstituting the emergence of the A.Br.081(Ames) polytomy in much greater detail and help understand how Germany imported only two among the nine branches of the polytomy [89]. A large number of Chinese isolates have been genotyped by MLVA or canSNP in the past, but usually not by WGS yet, which is necessary to achieve the required phylogenetic depth and precision [74,80,92,98,99].

New data from numerous countries, previously under-sampled, or still lacking WGS data, will allow researchers to (re-)evaluate and possibly challenge the model proposed here. An ancient presence of *B. anthracis* in Asia cannot be entirely ruled out since the most basal C clade has until now been found only in North America. The presence of the *B. anthracis* C clade in North America could be ancient, and in this case, Asia would be a likely source. Alternatively, it could be the result of a recent import of unknown geographic origin, as illustrated in the present work by the A.Br.Karnataka lineage present in India, which was initially identified by a unique strain isolated in North America.

## Data Availability

The genome sequence data presented in this study are available from the NCBI database under the BioProject ID: PRJNA309927. These and the accession numbers of publicly available genome sequences analyzed are listed in the Supplementary Materials (Supplementary Table S1) of this study.

## Supplementary Materials

The following supporting information can be downloaded at: https://www.mdpi.com/article/10.3390/pathogens14101041/s1, Table S1: List of assemblies and SRA accession numbers, and metadata. Table S2: List of SNPs called among the 80 strains used in Figure 2, together with the genotype of hypothetical nodes within the Maximum Parsimony Tree, and the corresponding Newick-format tree.

## Abbreviations

The following abbreviations are used in this manuscript:

VNTR	Variable Number of Tandem Repeats
MLVA	Multiple Locus VNTR Analysis
ANI	Average Nucleotide Identity
MLST	Multi-Locus Sequence Typing
ST	Sequence Type
SNP	Single Nucleotide Polymorphism
canSNP	Canonical SNP
WGS	Whole-Genome Sequence
wgSNP	Whole-Genome SNP Analysis
SRA	Sequence Reads Archive
NCBI	National Center for Biotechnology Information
EBI	European Bioinformatics Institute
ENA	European Nucleotide Archive
CE	Common Era
MRCA	Most Recent Common ancestor
ybp	Years Before Present
